# Pharmacist-Led Flu Vaccination Services in Romanian Community Pharmacies: Barriers, Perceptions, and Implementation Challenges

**DOI:** 10.3390/pharmacy14010036

**Published:** 2026-02-12

**Authors:** Marius Calin Chereches, Mihaela Simona Naidin, Alexandra Grosan, Radu Antoniu Patrascu, Anca-Maria Capraru, Marina Daniela Dimulescu, Adina Turcu-Stiolica

**Affiliations:** 1 Marketing, Management and Sustainability Department, Faculty of Pharmacy, George Emil Palade University of Medicine, Pharmacy, Science, and Technology of Targu Mures, 540142 Targu Mures, Romania; mariuscalinchereches@mac.com; 2 Department of Pharmaceutical Marketing and Management, Faculty of Pharmacy, University of Medicine and Pharmacy of Craiova, 200349 Craiova, Romania; adina.turcu@umfcv.ro; 3 Department of Pharmacology and Clinical Pharmacy, Faculty of Pharmacy, George Emil Palade University of Medicine, Pharmacy, Science, and Technology of Targu Mures, 540142 Targu Mures, Romania; 4 Doctoral School, University of Medicine and Pharmacy of Craiova, 200349 Craiova, Romania; patrascu.raduantoniu@gmail.com (R.A.P.); anca.mitran@umfcv.ro (A.-M.C.); marina.dimulescu@umfcv.ro (M.D.D.)

**Keywords:** community pharmacy, flu vaccination, pharmacist role, implementation barriers, health policy, Romania

## Abstract

Although pharmacist-led vaccination is a global standard for expanding immunization coverage, its adoption in Romania remains at an early stage. While previous studies have focused on early adopters, this research evaluates barriers, perceptions, and readiness among community pharmacies that do not yet provide this service, thereby addressing a critical knowledge gap regarding the “non-vaccinating” majority. A cross-sectional mixed-methods study was conducted among 208 pharmacists representing national chains, regional networks, and independent pharmacies. Quantitative data were analyzed using Chi-square tests and Spearman correlations to identify structural disparities, while a thematic analysis was employed to explore qualitative insights related to professional identity and operational barriers. We identified a clear mismatch between pharmacies’ willingness to provide vaccination services and their practical ability to implement them. Independent pharmacies demonstrated a strong intention to adopt vaccination services (71.4%) but were limited by financial constraints, with high implementation costs identified as a significant barrier (*p* = 0.014). In contrast, national pharmacy chains had sufficient resources yet faced marked staff resistance, with 43.9% reporting extreme reluctance (*p* = 0.038). These chains were concentrated in the capital region (*p* = 0.002), thereby positioning other pharmacies as key providers in underserved areas. Furthermore, thematic analysis revealed a deep-seated “professional identity” crisis, in which pharmacists struggle with the transition from medication specialists to clinical practitioners. The expansion of vaccination services cannot rely on a “one-size-fits-all” strategy. Successful national implementation requires a segmented policy approach, including financial subsidies to support independent pharmacies, change management strategies to engage the corporate workforce, and targeted regulatory education for regional networks to prevent vaccination deserts.

## 1. Introduction

Globally, the literature highlights a progressive expansion of the role of the community pharmacist in the provision of vaccination services, with implementations varying according to the national context and regulatory framework. Pharmacists are described as easily accessible and strategically positioned health professionals in the community, able to contribute to improving population access to immunization throughout the life course. Countries with well-established health systems, such as Australia, Canada, France, Portugal, the United Kingdom and the United States, have integrated vaccination into community pharmacies as part of routine services, including vaccines against viruses such as influenza, COVID-19, pneumococcal, HPV, herpes zoster and, more recently, the respiratory syncytial virus (RSV) vaccine [1]. Studies conducted primarily in the United States show that the involvement of community pharmacies in vaccination, through the roles of educator, facilitator and immunizer, is associated with increases in immunization rates, although the effectiveness of this model is influenced by legislative factors, reimbursement mechanisms and technological infrastructure [2]. Evidence synthesized at an international level indicates that pharmacist interventions have a positive impact on vaccination coverage, regardless of the type of intervention, supporting the relevance of this model in public health [3,4]. The COVID-19 pandemic has been a major factor in accelerating and validating the role of the pharmacist in immunization, highlighting both the potential of community pharmacies as accessible vaccination points and the significant differences in implementation between countries, determined by legislative, economic and social context [5,6].

The benefits of providing vaccination services through community pharmacies are consistently demonstrated in the literature, with a positive impact on population access to immunization and patient experience. Studies show that community pharmacies contribute to increasing the accessibility of vaccination services through their proximity to the community and the integration of vaccination into the routine operations of pharmacies, thereby facilitating access for individuals who have difficulty accessing traditional medical services [7,8]. At the same time, vaccination in pharmacies is associated with high levels of patient satisfaction, with patients evaluating positively both the information received and the vaccination procedure, including its safety [9]. Data from Germany indicate that a significant proportion of people vaccinated in pharmacies would not have sought vaccination in another healthcare setting, suggesting that this model contributes to attracting previously unvaccinated population segments [10].

The high acceptability of this service and the intention of patients to return for future vaccinations reflect a strong level of trust in pharmacists, who are perceived as competent and accessible health professionals, capable of playing a key role in promoting vaccination and reducing access inequities, particularly among vulnerable populations [7,11]. The implementation of vaccination services through community pharmacies varies significantly between countries and is strongly influenced by the regulatory framework and the scope of practice granted to pharmacists. In some contexts, such as certain European countries, pharmacy vaccination services are well-established and integrated into public health strategies, with pharmacists recognized as essential providers of influenza vaccines and other preventable diseases, based on positive experiences even prior to the COVID-19 pandemic [12]. In other regions, however, these services remain at an earlier stage of development, having been recently introduced or progressively expanded through legislative changes. For example, in the province of Quebec, the authority of pharmacists to administer vaccines was expanded in 2020, creating significant opportunities to increase access, while also generating persistent challenges related to the organization and the equitable distribution of access [13].

These differences in regulation and scope of practice are reflected in disparities in the adoption of vaccination in pharmacies and in variable levels of acceptance and service utilization. Studies indicate that where vaccination in pharmacies is well supported and organized, there is a high level of stakeholder support and clear patient benefits; however, barriers such as out-of-pocket costs and service organization constraints may limit the uniform expansion of the model [14]. In this context, expanding the categories of personnel authorized to vaccinate and increasing the availability of publicly funded vaccines in pharmacies are considered essential strategies to improve vaccination accessibility and acceptability [15]. Although influenza remains a major public health concern and pharmacists have significant potential to expand vaccination programs, their actual involvement in immunization remains limited. Numerous practical barriers persist resulting in many pharmacists not fully assuming this role, and continued reliance on conventional vaccination pathways, thereby contributing to suboptimal vaccination coverage [16,17].

Pharmacists have the professional training, physical accessibility and professional responsibility to promote vaccination, particularly given their frequent contact with patients who may not regularly consult physicians [18,19]. However, one factor contributing to their limited involvement in vaccination activities is insufficient immunization training within undergraduate pharmacy curricula as reflected in studies reporting suboptimal knowledge levels and the persistence of misperceptions regarding vaccine safety and promotion [20,21]. Reducing vaccine hesitancy is a central element of increasing immunization rates, and pharmacists, through their roles as educators, facilitators, and vaccine providers, are well positioned to support more favorable public attitudes toward immunization [22].

Romania introduced pharmacy-based influenza vaccination in October 2022 as part of eleven newly defined advanced pharmaceutical services, requiring specific certification through university-based training programs [23]. Despite favorable regulatory changes including partial reimbursement for eligible populations [24], adoption has remained limited: as of early 2024, only 442 pharmacies (~7% of Romania’s 6500 community pharmacies) were authorized to vaccinate, with geographic clustering in Bucharest and major urban centers [25]. Previous research examined early adopters and found high patient satisfaction. However, the vast majority of pharmacies (>93%) do not yet offer vaccination services due to concerns regarding remuneration [25]. Understanding the barriers that prevent this non-vaccinating majority of pharmacists from participating in vaccination programs is essential to achieving equitable national coverage and addressing Romania’s historically low influenza vaccination rates. The primary objective of this study is to assess the characteristics and perspectives of pharmacies that do not provide vaccination services, given that only a small proportion are currently authorized, thereby highlighting a significant knowledge gap and a marked discrepancy in service implementation.

## 2. Materials and Methods

### 2.1. Study Design and Data Collection

A cross-sectional study was conducted using data collected from pharmacists working in pharmacies that do not offer influenza vaccination services in Romania. Data were collected via an online, self-administered, anonymous survey on the Google Forms platform (Supplementary File S1).

The research was designed for community pharmacies that do not have authorization for the influenza vaccination service. Pharmacies that already offer influenza vaccination services and hospital pharmacies were excluded from this survey.

All community pharmacies in Romania (approximately 6500) were eligible for inclusion, and pharmacists in charge or pharmacy managers were invited to complete the questionnaire. Participants were recruited using a multi-channel approach. Invitations to complete the questionnaire were sent through local branches of the College of Pharmacists (the national professional regulatory body in Romania), professional pharmacy associations, regional or national pharmacy chains, or social media groups for pharmacies. As there is no centralized, publicly accessible registry or email database of community pharmacists, a non-probability snowball sampling approach was employed. This approach was used to maximize outreach within a hard-to-reach professional population and does not imply statistical representativeness of the sample. The strategy facilitated the inclusion of both independent pharmacists and those working within large corporate structures, while ensuring that individual responses remained independent and anonymous.

The questionnaire has been developed internally by the research team based on findings from a previous study [25], a literature review, and the current pharmaceutical legislative framework. It was piloted with a small group of pharmacists to ensure clarity of wording and to confirm that completion time did not exceed 15 min. The questionnaire comprised 31 items, including 27 closed-ended questions (multiple choice, Likert scales, and checkboxes) and 4 open-ended questions. Items were organized into six sections: general information and demographics; influenza vaccination services in pharmacies; resources and infrastructure; regulatory processes; perceptions and attitudes (including economic and financial factors); future plans; and a final section consisting of four open-ended questions exploring additional insights.

The regional geographic distribution of the participating pharmacies was recorded to ensure national coverage. This is particularly relevant in the Romanian context, where demographic criteria (population-to-pharmacy ratios) are strictly enforced in urban areas but waived in rural areas, leading to an uneven distribution of pharmaceutical services. Analyzing all the development regions allowed the study to account for systemic variations in pharmacy organization and accessibility.

This study was conducted in accordance with the Declaration of Helsinki and approved by the Ethics Committee of the University of Medicine and Pharmacy of Craiova, Romania (no. 239/20.06.2025). All data was collected anonymously.

### 2.2. Analytic Methods

Data collection and initial validation were performed using the Google Forms platform, after which the database was exported and coded for statistical processing. Descriptive statistics were computed to summarize demographic characteristics and categorical survey responses, with results expressed as absolute frequencies (*n*) and relative percentages (%).

To evaluate the associations between the types of pharmacies (National = national pharmacy chains, Regional = regional pharmacy chains, Independent = independent pharmacies) and various categorical variables (e.g., geographic distribution, regulatory knowledge, implementation intent, and perceived barriers), Pearson’s Chi-square test (χ^2^) was employed. When the theoretical assumptions underlying the Chi-square test were not met (specifically when expected cell frequencies were below 5), Fisher’s Exact Test was utilized to ensure the robustness of the inferential analysis.

To assess the strength and direction of relationships between ordinal variables (such as Likert scale responses regarding staff reluctance, profitability perception, and legal concerns), Spearman’s rank correlation coefficient (rho, ρ) was calculated. This non-parametric approach was selected to appropriately account for the ranked nature of the data.

All statistical tests were two-tailed, and a *p*-value < 0.05 was considered statistically significant. Quantitative analyses was conducted using R version 4.3.1, while qualitative data processing and thematic coding were facilitated by the Delve tool. Qualitative data were analyzed using a thematic analysis framework involving three stages: (1) open coding to identify initial concepts, (2) thematic coding to group related codes into categories, and (3) conceptualization to define overarching themes. Two researchers independently coded the responses, and discrepancies were resolved through discussion until consensus was reached [26].

Given the broad range of variables examined, these statistical analyses should be interpreted as exploratory, aimed at identifying key trends and informing targeted policy interventions.

## 3. Results

### 3.1. Characteristics of Participating Pharmacies

The study included a total of 208 community pharmacies that do not currently offer influenza vaccination services (*N* = 208). The sample was categorized according to pharmacy-organization-type national chains, which constituted the majority of the sample (*N* = 148); regional chains (*N* = 18); and independent pharmacies (*N* = 42).

Analysis of geographic distribution revealed a statistically significant association between pharmacy type and development region (*p* = 0.002). National chain pharmacies were highly concentrated in the Bucharest-Ilfov region, accounting for 25.7% (*n* = 38) of the national chain respondents. Additional concentrations were observed in the Center region (17.6%, *n* = 26) and the West region (10.1%, *n* = 15). In contrast, regional chains were predominantly located in the South-West region, which represented 44.4% (*n* = 8) of this subgroup. The North-West region also showed a notable presence of regional chains (16.7%, *n* = 3). Independent pharmacies appeared to be most prevalent in the Center region, representing 33.3% (*n* = 14) of the independent respondents. They also demonstrated a substantial presence in the South-West (21.4%, *n* = 9) and South-East (9.5%, *n* = 4) regions. Overall, the data highlights structural disparities in pharmacy distribution across development regions, with national chains clustering in the capital and major economic hubs, while regional and independent pharmacies provide essential coverage in other regions, particularly the South-West and Center, as presented in Table 1.

### 3.2. Perceptions, Barriers, and Staff Attitudes

The survey identified significant disparities in how different pharmacy types perceive the implementation of influenza vaccination services, particularly with respect to regulatory knowledge, future intent, and operational barriers, as presented in Table 2, Table 3, Table 4 and Table 5.

#### 3.2.1. Regulatory Awareness and Implementation Intent

A statistically significant association was observed between pharmacy type and knowledge of the authorization process (*p* < 0.001). National chains demonstrated high awareness, with 66.2% of respondents reporting that they had been informed about the process. Independent pharmacies showed similarly high levels of awareness (61.9%). In contrast, regional chains exhibited a notable knowledge gap; 44.4% of respondents from regional networks reported not been informed about the authorization process, compared to only 8.8% in national chains and 16.7% in independent pharmacies, as presented in Table 2.

Regarding the intention to implement the service (*p* < 0.001), independent pharmacies expressed the highest direct interest, with 71.4% indicating that they were considering implementation. In contrast, only 28.4% of respondents from national chains reported a specific intention for their unit to implement the service. This finding may be related to the fact that 37.2% indicated that other pharmacies within their chain already provide vaccination.

#### 3.2.2. Perceived Barriers

The types of barriers (financial and competitive concerns, staff reluctance) identified varied significantly by pharmacy structure. Independent pharmacists were significantly more likely to cite “High implementation costs” (21.4%) as a barrier compared to their counterparts in national chains (5.4%) (*p* = 0.014). Similarly, fear of “Competition from other providers” was higher among independents (11.9%) than national chains (2.0%) (*p* = 0.041).

Variation in staff attitudes was also statistically significant (*p* = 0.029). National chains reported the highest levels of resistance, with 43.9% of respondents describing their staff as “Extremely reluctant.” In contrast, 16.7% of independent pharmacies reported extreme reluctance.

#### 3.2.3. Workforce Willingness to Train

Willingness to undergo vaccination training differed significantly across pharmacy types (*p* = 0.029). Independent pharmacists demonstrated the highest willingness (59.5%), followed by regional chains (50.0%), whereas pharmacists in national chains reported the lowest willingness (30.4%).

#### 3.2.4. Operational and Regulatory Barriers

The authorization process presented challenges that were reported consistently across pharmacy types (*p* > 0.05), as presented in Table 3. The most frequently identified barrier was the lack of adequate physical space or the strict spatial requirements. This was reported by 69.6% of national chain respondents, 72.2% of regional chain respondents, and 59.5% of independent pharmacists (*p* = 0.568). Bureaucratic requirements were also frequently reported. The “Required documentation” was the second most significant barrier, particularly for national chains (50.7%) and regional chains (55.6%). Meeting equipment standards was also a major concern, cited by nearly half of the respondents in national chains (46.6%).

#### 3.2.5. Legal Liability and Competency Fit

Pharmacists reported substantial concern regarding the legal implications of administering vaccines. When asked about “Fear of legal liability,” a significant portion of respondents indicated they were concerned “To a great extent” (National: 48.0%; Regional: 44.4%; Independent: 38.1%). This level of concern did not differ significantly across pharmacy types (*p* = 0.727), suggesting a systemic need for clearer legal protection or indemnification for vaccinating pharmacists.

Opinions regarding whether vaccination aligns with current pharmacist competencies were mixed. Only 27.7% of national chain pharmacists and 35.7% of independent pharmacists considered the service as “Completely appropriate,” whereas 23.0% of national chain pharmacists and 19.0% of independent pharmacists viewed it as “Not appropriate”.

#### 3.2.6. Financial Perceptions and Profitability

The economic viability of the service remains a contentious issue, characterized by skepticism and a clear divide in perceived financial barriers.

There is a statistically significant disparity regarding the financial burden of setting up the service (*p* = 0.014). Nearly a quarter (21.4%) of independent pharmacists cited “High implementation costs” as a missing resource. Conversely, only 5.4% of national chain respondents viewed costs as a barrier, reflecting the stronger financial capacity of corporate networks.

Overall, pharmacists expressed pessimism regarding the direct financial returns of vaccination services (*p* = 0.695, referring to the profitability expectations across the three groups perceptions). Profitability expectations were generally low, with the plurality of respondents across all groups indicating that the service would be only “Slightly profitable” (National: 31.1%; Regional: 27.8%; Independent: 21.4%). A negative outlook was also observed: 33.3% of independent pharmacists considered the service “Not profitable at all,” compared to 19.6% in national chains. A high degree of uncertainty persisted, with approximately one-quarter of respondents (e.g., 26.4% of national chains) stating they “Cannot estimate” profitability, indicating limited clarity regarding business models or reimbursement mechanisms for this service and lack of clear business models or reimbursement clarity for this new service.

Regarding financial sustainability, the survey assessed the pharmacists’ preferred payment models. The analysis compared three potential funding mechanisms: full reimbursement by the Health Insurance House (HIH), partial reimbursement by the HIH, and full payment by the patient. The results indicate a strong consensus across the profession, regardless of the pharmacy’s organizational structure. The vast majority of respondents favored full reimbursement by HIH. This option was preferred by 66.9% (*n* = 99) of pharmacists in national chains, 72.2% (*n* = 13) in regional chains, and 78.6% (*n* = 33) in independent pharmacies. Partial reimbursement was supported by 17.6% of national chain respondents, while a fully private-pay model (“Fully paid by the patient”) was the least favored option, selected by only 15.5% of national chain pharmacists and 9.5% of independent pharmacists. Across all organizational structures, respondents indicated that the viability of the vaccination service depends primarily on public funding rather than out-of-pocket payments. Respondents also expressed similar expectations regarding the appropriate service fee, proposing a range of 35–50 RON (approximately 7–10 Euro) per vaccination.

Pharmacists across all practice settings demonstrated substantial agreement regarding perceived patient preferences for vaccination locations. No statistically significant differences were observed between national, regional, or independent pharmacies for any of the preference categories (*p* > 0.05), suggesting a uniform professional perception of patient behavior. The majority of respondents indicated that patients primarily prefer to be vaccinated “At the family doctor”, with response rates ranging from 83.3% (regional chains) to 88.1% (independent pharmacies). In contrast, the perception that patients prefer to be vaccinated “In the pharmacy” was low, selected by only 15.5% of national chain pharmacists, 16.7% of regional chain pharmacists, and 14.3% of independent pharmacists (*p* = 0.943). A minority of respondents perceived a preference for hospitals or clinics, reported slightly more frequently by regional chain pharmacists (22.2%) than by national chain pharmacists (10.8%), although this difference was not statistically significant (*p* = 0.288).

#### 3.2.7. Facilitators for Implementation

To identify potential levers for expanding vaccination services, respondents were asked to select the factors that would motivate them to implement the service. The analysis revealed statistically significant differences between pharmacy types, particularly regarding financial incentives and educational needs. A significant difference was observed regarding the reimbursement of the vaccination service by the HIH (*p* < 0.001). Among independent pharmacy owners, this is the paramount facilitator, with 69.0% identifying HIH reimbursement as a decisive factor. In stark contrast, less than one-third of respondents from national chains (29.1%) and regional chains (27.8%) considered this a primary driver.

The need for “Financial support for the initial investment” also differed significantly (*p* = 0.032). This factor was selected by 33.3% of regional chains and 31.0% of independent pharmacies, compared with 14.2% of national chain respondents.

In contrast, preference for “Higher rates for the vaccination service” (fee-for-service increase) did not differ significantly across groups (*p* = 0.881), being selected by approximately 37–39% of respondents regardless of pharmacy type. Educational needs also varied significantly (*p* = 0.032), although in the opposite direction. The respondents from regional chains expressed the highest need for “More information and training” (33.3%). A moderate portion (20.3%) from national chains also cited training as a facilitator. Only 4.8% of independent pharmacists selected training as a necessary driver, suggesting a high level of self-perceived readiness.

Regarding market factors, “Increased demand from patients” was reported more frequently by national chains (40.5%) than by regional chains (16.7%), though this difference did not reach statistical significance (*p* = 0.165). Similarly, the “Simplification of the authorization process” did not differ significantly across pharmacy types (*p* = 0.165), although it was selected more frequently by independent pharmacists (28.6%) than by national chains (14.9%).

### 3.3. Correlations Between Perceptions and Barriers

**Figure 1:**
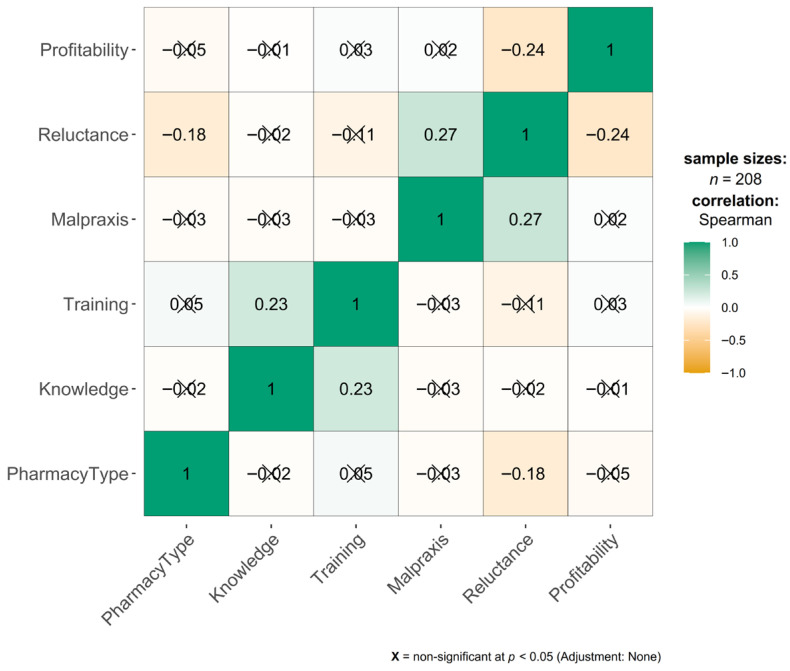
Correlation heatmap for non-parametric statistic variables.

There is a positive correlation between the presence of malpractice insurance coverage and staff reluctance (rho = 0.27, *p* < 0.001). Pharmacies with a higher proportion of staff who completed the mandatory influenza vaccination certification demonstrate significantly lower levels of professional hesitation, as illustrated in Figure 1. Trained pharmacists are less likely to identify “lack of skill” or “fear of adverse reactions” as primary barriers, instead emphasizing logistical or financial constraints. A moderate negative correlation was identified between perceived profitability and staff reluctance on vaccination service (rho = −0.24, *p* < 0.001).

**Figure 2:**
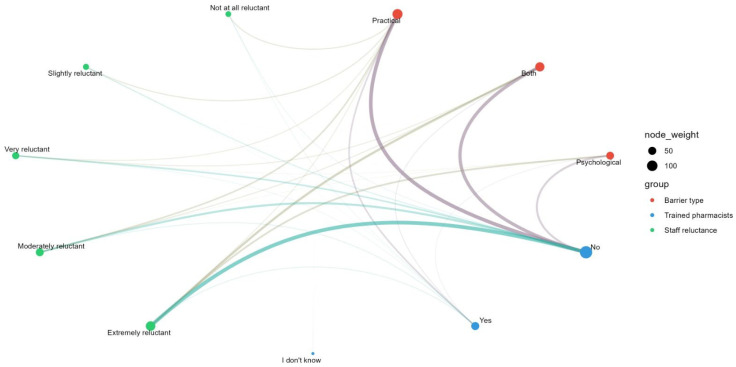
Associations between staff reluctance, the presence of trained pharmacists, and the type of barriers to providing vaccination services in community pharmacies in Romania.

Additionally, a positive association was observed between logistical barriers (e.g., lack of private counseling rooms, inadequate cold chain storage) and staff reluctance, as illustrated in Figure 2. When the physical environment is not adequately equipped, staff report higher levels of stress and resistance to providing the service.

### 3.4. Qualitative Thematic Analysis

Qualitative data showed how barriers varied across various pharmacy types and practice environments, while qualitative analysis offered a deeper understanding of the underlying perceptions and contextual factors. The themes are organized conceptually rather than numerically, emphasizing their interconnected nature. The analysis of open-ended responses identified five main themes presented in conceptual sequence: professional identity as the primary barrier; economic, legislative, and operational factors as emergent barriers; and limited public awareness as a consequential barrier to pharmacy-based influenza vaccination.

**Theme 1: Professional identity, trust and role boundaries**

The central tension concerns whether vaccination falls within the professional scope of pharmacy practice (is part of a pharmacist’s work), with several respondents viewing this activity as outside their defined role. Many described the pharmacist primarily as a “medication specialist,” rather than a “medical practitioner” (they identify the pharmacist profession as “medication specialist”, not “medical practitioner”).

*I disagree with vaccination in community pharmacies. I chose the profession of pharmacist, not that of a general practitioner or doctor. Moreover, it seems to me an insult to us as specialists in medicinal products.*

This perspective reflects a broader concern regarding the potential dilution of professional identity and perceived role encroachment. Respondents suggested that patients may place greater trust in general practitioners and other medical staff for vaccination, as these professionals are viewed as legitimate authorities for clinical procedures. Some participants also indicated that patients may prefer vaccination by general practitioners to avoid disrupting established relationships, particularly given that vaccination represents a source of revenue for medical providers. Participants further noted that patients do not trust pharmacists to administer the influenza vaccine, given their prevailing perception of pharmacists as suppliers of medicines rather than providers of healthcare services. Resistance to pharmacy-based influenza vaccination was also linked to concerns about professional competition with general practitioners and other healthcare providers, including fears of overstepping professional boundaries and potentially affecting collaborative relationships. Conversely, other respondents argued that pharmacists should be seen as equals to doctors as healthcare professionals, taken seriously, and therefore able to provide healthcare services such as flu vaccination, and therefore properly valued.

Some participants additionally highlighted that pharmacy-based influenza vaccination may represent a particularly appropriate solution in rural areas with limited access to medical services, where such services could address existing care gaps.

**Theme 2—Economic and financial constraints**

The lack of adequate compensation—or the absence of reimbursement—for influenza vaccination services is perceived as a significant barrier. Pharmacists emphasized the need for this service to be reimbursed by the Health Insurance House at a level comparable to that of general practitioners. They also indicated that remuneration should be directed to the pharmacist who administers the vaccine rather than to the legal entity operating the pharmacy.

*The appropriate payment for the pharmaceutical service is between 50 and 100 lei/vaccination. The payment should go to the pharmacist who performed the vaccination, not to the employer.*

Reimbursement at a level equivalent to that provided for general practitioners was framed not only as a financial consideration but also as a matter of professional recognition and parity. The current model in which pharmacies collect payments from patients was described as financially unsustainable and inequitable, particularly in light of the level of professional responsibility involved.

Participants also noted that the required investments for commissioning the vaccination space are significant and should be supported, at least in part, through external funding sources rather than relying solely on pharmacy resources.

**Theme 3—Legislative and administrative burden**

Some participants perceive the authorization process as complex, time-consuming and characterized by excessive requirements and called for greater simplification and digitalization. Respondents indicated that a comprehensive guideline outlining both the authorization procedure and the practical aspects of delivering influenza vaccination services in pharmacies would be beneficial. Fear of legal consequences in the event of adverse reactions to vaccination was frequently mentioned, reflecting concerns about unclear boundaries of professional responsibility and the need for clearer liability protection.

*Nothing should happen legally to the person administering the vaccine if the patient has an adverse reaction.*

**Theme 4—Knowledge, training and operational preparedness**

Participants identified limited access to training and operational constraints as practical barriers to implementation. The required certification training is currently available only in university centers, limiting participation for pharmacists who are unable to travel.

Some respondents expressed concerns that pharmacists’ knowledge of anatomy and physiology is insufficient to ensure confidence in managing potential adverse reactions, particularly anaphylaxis.

Operational constraints were also emphasized, including limited time and insufficient staffing. Many pharmacists reported being overwhelmed by administrative responsibilities, leaving little capacity to incorporate vaccination services, especially in single-pharmacist settings. The current physical layout of pharmacies was described as offering limited flexibility to add the space for vaccination, as legally required, and cannot ensure patient privacy.

When asked what would facilitate implementation, respondents suggested simplification of spatial requirements and integration of vaccination training into undergraduate pharmacy curricula.

*(What would help?) Simplification of space requirements. Introduction of qualification into the curriculum of pharmacy faculties.*

**Theme 5—Public awareness and the information deficits**

Participants indicated that many patients are unaware that pharmacies can provide influenza vaccination, attributing this to the absence of public information campaigns. Respondents emphasized the need for increased media promotion and public communication efforts. In addition, general vaccination hesitancy, fueled by misinformation and limited public education regarding immunization, was perceived as contributing to low demand for vaccination in pharmacies.

*Encouraging the population to get vaccinated because there is a lot of misinformation regarding vaccination in general and people choose not to get vaccinated for completely absurd reasons.*

This theme emerged not as a primary barrier, but rather as a downstream consequence of limited implementation: the small number of pharmacies offering vaccination services reduces public visibility, which in turn reinforces low awareness among the population.

Collectively, these findings underscore not only operational challenges but fundamental questions about professional roles, trust, and the evolving role of community pharmacists in Romania’s healthcare system. The conceptual progression—from core identity concerns through practical barriers and awareness gaps—suggests that expanding vaccination services needs interventions across multiple levels, including clarification of professional responsibilities, societal engagement and supportive economic and regulatory measures.

## 4. Discussion

This study provides the first detailed assessment of the implementation readiness of Romanian community pharmacies that do not currently provide vaccination services. By situating our results within the international literature, we identified both widely shared operational barriers to pharmacy-based vaccination (PBV) and challenges specific to the Romanian professional and market context.

Across settings, logistical constraints remain a consistent barrier to PBV implementation. In our study, “lack of adequate physical space” was the dominant barrier, cited by 69.6% of national chains and 59.5% of independent pharmacies. This aligns with findings from the Middle East and Jordan, where Jarab et al. identified “lack of space for storage” (74.1%) and “lack of collaboration with other healthcare professionals” (85.6%) as top-tier barriers [27]. Similarly, research in Lebanon by Youssef et al. demonstrated that pharmacists who perceived space and staffing as barriers were significantly less likely to implement the service (OR = 0.410) [28]. The ubiquity of this issue suggests that the rigid pharmaceutical architecture norms—often designed for dispensing rather than clinical services—are a global impediment requiring regulatory flexibility [29].

Beyond these shared barriers, our results reveal a clear mismatch between motivation and capacity across pharmacy types. Independent pharmacists were significantly more willing to train (59.5%) and implement the service (71.4%) than their counterparts in national chains. This resonates with the findings of Youssef et al., who observed that pharmacy owners were 1.5 times more likely to be willing to vaccinate compared to employee pharmacists (OR = 1.504). This suggests that for independent owners, vaccination is a strategic business differentiator and a pathway to professional autonomy [28].

In contrast, the high “staff reluctance” we observed in national chains (43.9% extremely reluctant) mirrors the “workload concerns” and “time pressures” reported in systematic reviews of chain-based practices [30,31]. In corporate settings, without direct financial incentives or workflow adjustments, the addition of clinical duties is often perceived merely as an increased burden rather than a professional opportunity [32].

Within the Romanian context, qualitative findings point to uncertainty regarding pharmacists’ evolving professional role. Vaccination was frequently perceived as being outside the traditional scope of practice, reflecting a persistent identification with a medication-focused role. The qualitative analysis identified professional identity as the main barrier, rather than solely economic or logistical issues. The tension related to identity appears more fundamental, especially in the Romanian context, where the question of being a “medication specialist” versus a “medical practitioner” needs to be addressed. These findings align with a recent paper by McKeirnan et al. [33]. This internal conflict is well-documented in the literature. Watson et al. describe this as a tension between the traditional “Dispenser” identity and the emerging “Clinician” or “Healthcare Provider” identity [34]. In systems where pharmacy services are well-established (e.g., UK, Canada), this identity has shifted; however, in emerging contexts like Romania, the “Dispensing Pharmacist” identity remains deeply rooted. Our results suggest that regulatory authorization alone may be insufficient and that workforce development beginning at the university level is needed to bridge this identity gap.

Perceived patient preferences also emerged as a significant barrier, with 85% of respondents believing that patients prefer general practitioners for vaccination. However, evidence from neighboring Poland suggests this may be a self-limiting belief rather than a market reality. A survey of Polish patients—who share a similar post-communist healthcare heritage—found that 94.5% of patients vaccinated in pharmacies were satisfied, and 98.5% felt safe, citing convenience and accessibility as key drivers. This indicates that while Romanian pharmacists fear a lack of patient demand, the “Field of Dreams” effect applies: if the service is built and accessible, patient trust follows [35]. The trust deficit is bidirectional: patients’ lack of trust affects pharmacists and pharmacies. The GP preference is not only about competence but also about the economic relationship. The use of flu vaccination services in pharmacies builds on trust and confidence, as documented in published papers [36,37].

The perception of limited patient trust identified in our study contrasts with international evidence. In established systems such as Germany, studies have reported high levels of patient satisfaction and trust in pharmacist-led vaccination, often reaching populations that do not regularly visit family physicians [10]. This suggests that the barriers perceived by Romanian pharmacists may be contextual and linked to the ‘novelty’ of the service. As pharmacist-led vaccination becomes more integrated into the national healthcare landscape and public exposure increases, these perceptions of trust are likely to evolve toward the higher levels seen in other European settings.

Finally, the demand for reimbursement (HIH coverage) was a uniform consensus in our study (66.9–78.6%), particularly for independent pharmacies (69.0%). This aligns with the global consensus that pharmacist-based vaccination sustainability is contingent on adequate remuneration. A systematic review of financial incentives confirms that in developing or economically stratified contexts, financial models are the strongest predictor of service uptake [5]. Without the “financial safety net” of reimbursement, the willingness of independent pharmacists—who are the most motivated but least capitalized—cannot translate into practice. Multilevel intervention is needed for full-scale implementation. Reimbursement is critical but insufficient alone [38].

This study has limitations inherent to its cross-sectional design. The use of a snowball sampling method may have introduced selection bias, potentially over-representing pharmacists who are more digitally active, and limits the generalizability of the findings beyond the study sample. This approach was chosen due to the absence of a centralized, publicly accessible sampling frame for community pharmacists. Additionally, the self-reported nature of the data reflects perceptions rather than objective operational audits. However, given the exploratory purpose of the study, these perceptions are critical determinants of current implementation behavior.

## 5. Conclusions

This study provides the first structural analysis of the barriers characterizing Romanian community pharmacies that do not yet provide influenza vaccination services. Our findings demonstrate that the path to national implementation is not obstructed by a single, uniform barrier, but rather by a complex and differentiated landscape that varies significantly by organizational type.

First, we identified a motivation–capacity imbalance. Independent pharmacies emerged as the most willing segment, displaying the highest intention to implement services and the greatest workforce willingness to train. However, they remain operationally constrained, particularly by high implementation costs and concerns regarding competition. Conversely, national chains possess the necessary financial resilience and logistical infrastructure but face severe internal inertia, reporting the highest levels of staff reluctance. This pattern suggests that although implementation willingness is concentrated in the independent sector, structural capacity is more pronounced within corporate networks.

Second, the barriers are not merely logistical but existential. The professional identity crisis—where pharmacists struggle to view themselves as clinical practitioners rather than medication specialists—remains a fundamental psychological hurdle. This is reinforced by the widespread perception that patients prefer family doctors for vaccination, which may discourage service expansion despite international evidence indicating strong patient acceptance of pharmacist-led vaccination A dual educational approach is required—integrating vaccination competencies into university curricula while also strengthening public awareness initiatives to support the pharmacist’s role as an immunizer.

Ultimately, the sustainability of pharmacy-based vaccination in Romania is contingent upon integrating the service into the National Health Insurance framework. A reimbursement model is not merely a financial preference but a prerequisite for equitable implementation, ensuring accessibility regardless of pharmacy organizational type.

## Figures and Tables

**Table 1:** Characteristics of the included pharmacies by type of pharmacy.

Variable	Total *N* = 208	National *N* = 148	Regional *N* = 18	Independent *N* = 42	*p*-Value
Environment					
Urban	170 (81.7%)	135 (91.2%)	13 (72.2%)	22 (52.4%)	<0.001
Rural	29 (13.9%)	7 (4.7%)	5 (27.8%)	17 (40.5%)	
Peri-Urban	9 (4.3%)	6 (4.1%)		3 (7.1%)	
Number of patients/day					
<100	50 (24%)	30 (20.3%)	6 (33.3%)	14 (33.3%)	0.072
100–300	130 (62.5%)	97 (65.5%)	8 (44.4%)	25 (59.5%)	
300–500	20 (9.6%)	17 (11.5%)	3 (16.7%)		
>500	8 (3.8%)	4 (2.7%)	1 (5.6%)	3 (7.1%)	
Number of pharmacists					<0.001
1	40 (19.2%)	22 (14.9%)	7 (38.9%)	11 (26.2%)	
1–3	151 (72.6%)	122 (82.4%)	9 (50.0%)	20 (47.6%)	
3–5	8 (3.8%)	1 (0.7%)	1 (5.6%)	6 (14.3%)	
>5	9 (4.3%)	3 (2.0%)	1 (5.6%)	5 (11.9%)	
Region					0.002
Bucharest-Ilfov	42 (20.2%)	38 (25.7%)	4 (22.2%)	-	
Center	42 (20.2%)	26 (17.6%)	2 (11.1%)	14 (33.3%)	
North-East	20 (9.6%)	14 (9.5%)	1 (5.6%)	5 (11.9%)	
North-West	21 (10.1%)	15 (10.1%)	3 (16.7%)	3 (7.1%)	
South	22 (10.6%)	17 (11.5%)		5 (11.9%)	
South-East	14 (6.7%)	10 (6.8%)		4 (9.5%)	
South-West	30 (14.4%)	13 (8.8%)	8 (44.4%)	9 (21.4%)	
West	17 (8.2%)	15 (10.1%)		2 (4.8%)	

**Table 2:** Regulatory awareness and implementation intent.

Variable	Total *N* = 208	National *N* = 148	Regional *N* = 18	Independent *N* = 42	*p*-Value
Knowledge on the legislation that allows pharmacies to provide flu vaccination services					0.216
Yes	145 (69.7%)	104 (70.3%)	10 (55.6%)	31 (73.8%)	
Yes, but limited	53(25.5%)	40 (27.0%)	5 (27.8%)	8 (19.0%)	
No	10 (4.8%)	4 (2.7%)	3 (16.7%)	3 (7.1%)	
Have you been informed about the authorization process for providing vaccination services?					<0.001
Yes	128 (61.5%)	98 (66.2%)	4 (22.2%)	26 (61.9%)	
Yes, but I do not remember	52 (25%)	37 (25.0%)	6 (33.3%)	9 (21.4%)	
No	28 (13.5%)	13 (8.8%)	8 (44.4%)	7 (16.7%)	
Have you considered implementing a flu vaccination service in your pharmacy?					<0.001
Yes	76 (36.5%)	42 (28.4%)	4 (22.2%)	30 (71.4%)	
We have other pharmacies with vaccination	57 (27.4%)	55 (37.2%)	2 (11.1%)	0 (0.0%)	
No	69 (33.2%)	46 (31.1%)	11 (61.1%)	12 (28.6%)	
We will provide it	6 (2.9%)	5 (3.4%)	1 (5.6%)	0 (0.0%)	
Expected demand for the vaccination service					0.564
Very high	14 (6.7%)	11 (7.4%)	0 (0.0%)	3 (7.1%)	
High	29 (13.9%)	16 (10.8%)	6 (33.3%)	7 (16.7%)	
Moderate	65 (31.3%)	47 (31.8%)	5 (27.8%)	13 (31.0%)	
Low	50 (24.0%)	34 (23.0%)	4 (22.2%)	12 (28.6%)	
Very low	50 (24.0%)	40 (27.0%)	3 (16.7%)	7 (16.7%)	
Have you received requests from patients for vaccination services in your pharmacy?					0.641
Yes, frequently	24 (11.5%)	17 (11.5%)	1 (5.6%)	6 (14.3%)	
Yes, occasionally	56 (26.9%)	38 (25.7%)	7 (38.9%)	11 (26.2%)	
Very rarely	54 (26.0%)	42 (28.4%)	6 (33.3%)	6 (14.3%)	
Never	67 (32.2%)	46 (31.1%)	3 (16.7%)	18 (42.9%)	
I do not know	7 (3.4%)	5 (3.4%)	1 (5.6%)	1 (2.4%)	
Patients prefer to get vaccinated (Multiple-choice) ^a^					
At the family doctor	178 (85.6%)	126 (85.1%)	15 (83.3%)	37 (88.1%)	0.882
In the hospital/clinic	27 (13.0%)	16 (10.8%)	4 (22.2%)	7 (16.7%)	0.288
In the pharmacy	32 (15.4%)	23 (15.5%)	3 (16.7%)	6 (14.3%)	0.943
I do not know	15 (7.2%)	10 (6.8%)	2 (11.1%)	3 (7.1%)	0.95
Future intentions to implement the flu vaccination service in the future					<0.001
Yes, in the next 6 months	12 (5.8%)	10 (6.8%)	0 (0.0%)	2 (4.8%)	
Yes, in the next year	7 (3.4%)	1 (0.7%)	0 (0.0%)	6 (14.3%)	
Yes, but much later	43 (20.7%)	23 (15.5%)	5 (27.8%)	15 (35.7%)	
No	83 (39.9%)	65 (43.9%)	7 (38.9%)	9 (21.4%)	
I do not know	65 (31.3%)	49 (33.1%)	6 (33.3%)	10 (23.8%)	
What would make you implement the vaccination service in your pharmacy? (Multiple-choice) ^a^					
Simplification of the authorization process	37 (17.8%)	22 (14.9%)	3 (16.7%)	12 (28.6%)	0.165
Financial support for the initial investment	40 (19.2%)	21 (14.2%)	6 (33.3%)	13 (31.0%)	0.032
Increased demand from patients	78 (37.5%)	60 (40.5%)	3 (16.7%)	15 (35.7%)	0.165
Higher rates for the vaccination service	76 (36.5%)	55 (37.2%)	7 (38.9%)	14 (33.3%)	0.881
Reimbursement of the service by HIH	77 (37.0%)	43 (29.1%)	5 (27.8%)	29 (69.0%)	<0.001
More information and training	38 (18.3%)	30 (20.3%)	6 (33.3%)	2 (4.8%)	0.032

HIH, Health Insurance House. ^a^ Percentages do not total 100% because respondents could choose multiple options.

**Table 3:** Operational and structural barriers.

Variable	Total *N* = 208	National *N* = 148	Regional *N* = 18	Independent *N* = 42	*p*-Value
Which resources do you consider to be missing or insufficient in your pharmacy for the implementation of the vaccination service?					
Lack of adequate physical space	110 (52.9%)	83 (56.1%)	10 (55.6%)	17 (40.5%)	0.306
Lack of qualified staff	96 (46.2%)	74 (50.0%)	6 (33.3%)	16 (38.1%)	0.306
High implementation costs	17 (8.2%)	8 (5.4%)	0 (0.0%)	9 (21.4%)	0.014
Complex authorization process	22 (10.6%)	13 (8.8%)	3 (16.7%)	6 (14.3%)	0.453
Staff reluctance to administer vaccines	78 (37.5%)	59 (39.9%)	9 (50.0%)	10 (23.8%)	0.193
Fear of legal liability	55 (26.4%)	42 (28.4%)	7 (38.9%)	6 (14.3%)	0.193
Lack of patient demand	35 (16.8%)	27 (18.2%)	3 (16.7%)	5 (11.9%)	0.625
Competition from other providers	8 (3.8%)	3 (2.0%)	0 (0.0%)	5 (11.9%)	0.041
Authorization perception					0.641
Relatively simple	22 (10.6%)	16 (10.8%)	0 (0.0%)	6 (14.3%)	
Moderately complex	61 (29.3%)	40 (27.0%)	5 (27.8%)	16 (38.1%)	
Very complex and bureaucratic	47 (22.6%)	34 (23.0%)	4 (22.2%)	9 (21.4%)	
I do not know the authorization details	78 (37.5%)	58 (39.2%)	9 (50.0%)	11 (26.2%)	
Which aspects of the authorization process do you consider to be significant barriers? (Multiple-choice) ^a^					
Required documentation	102 (49%)	75 (50.7%)	10 (55.6%)	17 (40.5%)	0.568
Space requirements	141 (67.8%)	103 (69.6%)	13 (72.2%)	25 (59.5%)	0.568
Equipment requirements	92 (44.2%)	69 (46.6%)	8 (44.4%)	15 (35.7%)	0.568
Time required to obtain authorization	2 (1.0%)	2 (1.4%)	0 (0.0%)	0 (0.0%)	0.664
Costs associated with the authorization process	89 (42.8%)	56 (37.8%)	10 (55.6%)	23 (54.8%)	0.383

^a^ Percentages do not total 100% because respondents could choose multiple options.

**Table 4:** Financial perceptions and remuneration.

Variable	Total *N* = 208	National *N* = 148	Regional *N* = 18	Independent *N* = 42	*p*-Value
Profitability					0.695
Very profitable	13 (6.3%)	10 (6.8%)	0 (0.0%)	3 (7.1%)	
Moderately profitable	34 (16.3%)	24 (16.2%)	5 (27.8%)	5 (11.9%)	
Slightly profitable	60 (28.8%)	46 (31.1%)	5 (27.8%)	9 (21.4%)	
Not profitable at all	46 (22.1%)	29 (19.6%)	3 (16.7%)	14 (33.3%)	
Cannot estimate	55 (26.4%)	39 (26.4%)	5 (27.8%)	11 (26.2%)	
The estimated costs of setting up a vaccination space and equipping it with the necessary equipment					0.283
<1000 RON	23 (11.1%)	18 (12.2%)	0 (0.0%)	5 (11.9%)	
1000–3000 RON	51 (24.5%)	33 (22.3%)	7 (38.9%)	11 (26.2%)	
3000–10,000 RON	51 (24.5%)	31 (20.9%)	4 (22.2%)	16 (38.1%)	
>10,000 RON	8 (3.8%)	6 (4.1%)	0 (0.0%)	2 (4.8%)	
I cannot estimate	75 (36.1%)	60 (40.5%)	7 (38.9%)	8 (19.0%)	
Optimal payment method					0.679
Fully covered by HIH	145 (69.7%)	99 (66.9%)	13 (72.2%)	33 (78.6%)	
Partially covered by HIH	34 (16.3%)	26 (17.6%)	3 (16.7%)	5 (11.9%)	
Fully paid by the patient	29 (13.9%)	23 (15.5%)	2 (11.1%)	4 (9.5%)	
What amount do you think would be appropriate for the remuneration of pharmacists for this service?					0.248
<20 RON/vaccination	13 (6.3%)	11 (7.4%)	0 (0.0%)	2 (4.8%)	
20–35 RON/vaccination	36 (17.3%)	22 (14.9%)	6 (33.3%)	8 (19.0%)	
35–50 RON/vaccination	86 (41.3%)	58 (39.2%)	6 (33.3%)	22 (52.4%)	
>50 RON/vaccination	15 (7.2%)	13 (8.8%)	0 (0.0%)	2 (4.8%)	
I do not know	58 (27.9%)	44 (29.7%)	6 (33.3%)	8 (19.0%)	

HIH, Health Insurance House.

**Table 5:** Workforce attitudes and professional identity.

Variable	Total *N* = 208	National *N* = 148	Regional *N* = 18	Independent *N* = 42	*p*-Value
Concerns about Legal Liability					0.727
Not at all	17 (8.2%)	13 (8.8%)	1 (5.6%)	3 (7.1%)	
To a small extent	21 (10.1%)	15 (10.1%)	0 (0.0%)	6 (14.3%)	
To some extent	59 (28.4%)	37 (25.0%)	7 (38.9%)	15 (35.7%)	
To a great extent	95 (45.7%)	71 (48.0%)	8 (44.4%)	16 (38.1%)	
I do not know	16 (7.7%)	12 (8.1%)	2 (11.1%)	2 (4.8%)	
Fit with pharmacists’ competencies					0.641
Completely appropriate for pharmacists	59 (28.4%)	41 (27.7%)	3 (16.7%)	15 (35.7%)	
Partially appropriate for pharmacists	62 (29.8%)	42 (28.4%)	8 (44.4%)	12 (28.6%)	
Appropriate for some pharmacists	44 (21.2%)	34 (23.0%)	2 (11.1%)	8 (19.0%)	
Not appropriate for pharmacists	35 (16.8%)	27 (18.2%)	3 (16.7%)	5 (11.9%)	
I do not know	8 (3.8%)	4 (2.7%)	2 (11.1%)	2 (4.8%)	
Barrier type					0.727
Practical	90 (43.3%)	62 (41.9%)	7 (38.9%)	21 (50.0%)	
Psychological	46 (22.1%)	36 (24.3%)	3 (16.7%)	7 (16.7%)	
Both	72 (34.6%)	50 (33.8%)	8 (44.4%)	14 (33.3%)	
Staff reluctance					0.038
Not at all reluctant	21 (10.1%)	17 (11.5%)	1 (5.6%)	3 (7.1%)	
Slightly reluctant	24 (11.5%)	12 (8.1%)	1 (5.6%)	11 (26.2%)	
Very reluctant	48 (23.1%)	30 (20.3%)	5 (27.8%)	13 (31.0%)	
Moderately reluctant	37 (17.8%)	24 (16.2%)	5 (27.8%)	8 (19.0%)	
Extremely reluctant	78 (37.5%)	65 (43.9%)	6 (33.3%)	7 (16.7%)	
Trained pharmacists in the pharmacy					0.565
Yes	50 (25%)	33 (22.3%)	3 (16.7%)	14 (33.3%)	
No	139 (66.8%)	110 (74.3%)	13 (72.2%)	26 (61.9%)	
I do not know	9 (4.3%)	5 (3.4%)	2 (11.1%)	2 (4.8%)	
Pharmacists in the pharmacy willing to train					0.038
Yes	79 (38%)	45 (30.4%)	9 (50.0%)	25 (59.5%)	
No	81 (38.9%)	65 (43.9%)	6 (33.3%)	10 (23.8%)	
I do not know	48 (23.1%)	38 (25.7%)	3 (16.7%)	7 (16.7%)	

## Data Availability

The data presented in this study are available on request from the corresponding author due to privacy and ethical reasons.

## Supplementary Materials

The following supporting information can be downloaded at: https://www.mdpi.com/article/10.3390/pharmacy14010036/s1, File S1: Questionnaire on Barriers to the Implementation of Flu Vaccination Services in Romanian Community Pharmacies.
