# Supplementary material for: Pharmacist-Led Flu Vaccination Services in Romanian Community Pharmacies: Barriers, Perceptions, and Implementation Challenges
Source: Pharmacy (Basel). 2026 Feb 12;14(1):36. doi: 10.3390/pharmacy14010036 (PMC12921818; doi:10.3390/pharmacy14010036)
Supplement: Supplementary file 1 [file pharmacy-14-00036-s001.zip › pharmacy-4102734-supplementary.pdf]

Chestionar privind barierele în implementarea serviciilor de vaccinare antigripală în farmaciile comunitare din România

Vă mulțumim pentru participarea dumneavoastră la această cercetare!

Scopul cercetării

Această cercetare își propune să identifice barierele în implementarea serviciului farmaceutic avansat de vaccinare antigripală în farmacii, cu scopul de a înțelege mai bine provocările cu care se confruntă profesioniștii din domeniu.

Criterii de participare

Această cercetare se adresează exclusiv farmaciilor care NU au implementat încă serviciul de vaccinare antigripală.

⚠️ **Important:** Dacă vă desfășurați activitatea într-o farmacie în care se realizează deja vaccinarea antigripală, vă rugăm să nu completați acest chestionar.

Instrucțiuni de completare

- Vă rugăm să completați un singur chestionar pe farmacie
- Timpul estimat de completare: aproximativ 10-15 minute
- Toate răspunsurile sunt confidențiale și vor fi utilizate exclusiv în scopuri de cercetare

Aspecte etice

Această cercetare a primit aprobarea Comisiei de Etică a UMF.....

Participarea la această cercetare este voluntară. Prin completarea chestionarului, vă exprimați acordul de a participa la studiu.

Vă mulțumim pentru timpul acordat și pentru contribuția dumneavoastră la îmbunătățirea serviciilor farmaceutice!

* Indicates required question

Skip to question 1Skip to question 1

Informații generale

1. Tipul farmaciei * Dropdown

Mark only one oval.

- ◯ farmacie independentă
- ◯ farmacie parte dintr-un lanț național
- ◯ farmacie parte dintr-un lanț local/regional (2-3 județe)

2. Mediul în care se află farmacie *

Dropdown

Mark only one oval.

- ( ) urban
- ( ) periurban (comună în zona metropolitană/suburbanul unui oraș)
- ( ) rural tradițional (comună, sat)

3. Regiunea în care funcționează farmacia *

Dropdown

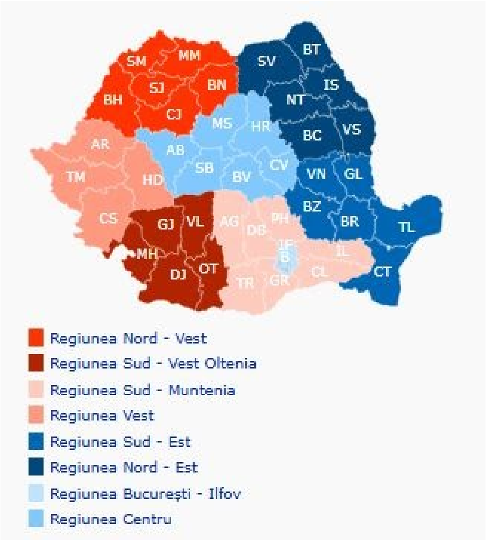

Mark only one oval.

- ( ) București -Ilfov
- ( ) Nord-Vest
- ( ) Sud-Vest Oltenia
- ( ) Sud-Muntenia
- ( ) Vest
- ( ) Sud-Est
- ( ) Nord-Est
- ( ) Centru

4. Număr aproximativ de pacienți deserviți zilnic (număr de bonuri) *

Dropdown

Mark only one oval.

- ( ) sub 100
- ( ) 100-300
- ( ) 300-500
- ( ) peste 500

5. Număr de farmaciști care lucrează în farmacie (inclusiv norme parțiale) * Dropdown

Mark only one oval.

- ⬭ 1
- ⬭ 2-3
- ⬭ 4-5
- ⬭ mai mult de 5

Skip to question 6

Informații despre serviciul de vaccinare antigripală în farmacii

6. Sunteți la curent cu legislația care permite farmaciilor să ofere servicii de vaccinare antigripală? * Dropdown

Mark only one oval.

- ⬭ Da, sunt pe deplin informat/ă
- ⬭ Da, dar am informații limitate
- ⬭ Nu, nu sunt la curent

7. Ați fost informat/ă despre procesul de autorizare pentru oferirea serviciilor de vaccinare? * Dropdown

Mark only one oval.

- ⬭ Da
- ⬭ Da, dar nu-mi mai amintesc detaliile
- ⬭ Nu

8. Ați luat în considerare implementarea serviciului de vaccinare antigripală în farmacia dvs.? * Dropdown

Mark only one oval.

- ⬭ Da, am analizat această posibilitate
- ⬭ Deja avem alte farmacii din lanțul nostru care sunt autorizate să vaccineze
- ⬭ Nu, nu am analizat această posibilitate
- ⬭ Suntem în proces de analiză

Skip to question 9

Resurse și infrastructură

9. Care dintre următoarele resurse considerați că lipsesc sau sunt insuficiente în farmacia dvs. pentru implementarea serviciului de vaccinare? (puteți selecta mai multe opțiuni) *

Check all that apply.

- ☐ Spațiu fizic adecvat pentru amenajarea unei zone de vaccinare
- ☐ Echipamente și materiale necesare (frigider special, materiale de protecție etc.)
- ☐ Personal calificat, suficient, pentru administrarea vaccinurilor
- ☐ Resurse financiare pentru investiția inițială
- ☐ Timp pentru gestionarea acestui serviciu suplimentar
- ☐ Other: ____________________

10. Există farmaciști în echipa dvs. care AU FĂCUT cursul obligatoriu pentru administrarea vaccinurilor? * Dropdown

Mark only one oval.

- Da
- Nu
- Nu știu

11. Există farmaciști în echipa dvs. care AR FI DISPUȘI SĂ URMEZE cursul obligatoriu pentru administrarea vaccinurilor? * Dropdown

Mark only one oval.

- Da
- Nu
- Nu știu

12. Estimați costul total pentru amenajarea unui spațiu de vaccinare și dotarea cu echipamentele necesare Dropdown

Mark only one oval.

- sub 1.000 lei
- 1.000-3.000 lei
- 3.000-10.000 lei
- peste 10.000 lei
- Nu pot estima

Skip to question 13

Reglementări și birocrație

13. Cum percepeți procesul de autorizare pentru serviciile de vaccinare? * Dropdown

Mark only one oval.

- Foarte complex și birocratic
- Moderat de complex
- Relativ simplu
- Nu cunosc detaliile procesului de autorizare

14. Care aspecte ale procesului de autorizare considerați că reprezintă bariere semnificative? (puteți selecta mai multe opțiuni) *

Check all that apply.

- [ ] Documentația necesară
- [ ] Cerințele privind spațiul
- [ ] Cerințele privind echipamentele
- [ ] Tipul necesar pentru obținerea autorizației
- [ ] Costurile asociate procesului de autorizare
- [ ] Other: ______________________________

15. Vă îngrijorează responsabilitatea juridică asociată cu administrarea vaccinurilor (malpraxis)? * Dropdown

Mark only one oval.

- ( ) În mare măsură
- ( ) Într-o oarecare măsură
- ( ) În mică măsură
- ( ) Deloc
- ( ) Nu știu/Nu m-am gândit

Skip to question 16

Percepții și atitudini

16. În ce măsură credeți că administrarea vaccinurilor se potrivește cu competențele farmacistului? * Dropdown

Mark only one oval.

- ( ) Este complet adecvat pentru farmaciști
- ( ) Este parțial adecvat pentru farmaciști
- ( ) Nu este adecvat pentru toți farmaciștii
- ( ) Nu este deloc adecvat pentru farmaciști
- ( ) Nu știu/Nu răspund

17. Care sunt PRINCIPALELE motive pentru care farmacia dvs. nu oferă în prezent servicii de vaccinare? (puteți selecta maximum 3 opțiuni) *

Check all that apply.

- [ ] Lipsa spațiului fizic adecvat
- [ ] Lipsa personalului calificat
- [ ] Costurile ridicate de implementare
- [ ] Procesul complex de autorizare
- [ ] Reticența personalului de a administra vaccinuri
- [ ] Teama de responsabilitate juridică
- [ ] Faptul că vaccinarea antigripală în farmacie nu este compensată de CNAS
- [ ] Lipsa cererii pentru servicii de vaccinare din partea pacienților
- [ ] Concurența din partea altor furnizori de servicii de vaccinare
- [ ] Nu considerăm că acest serviciu ar fi profitabil
- [ ] Other: ______________________________

18. În ce măsură există reticență din partea personalului farmaciei în ceea ce privește administrarea vaccinurilor? *

Mark only one oval.

	1	2	3	4	5	
Nici	◯	◯	◯	◯	◯	Reticență foarte mare

19. Considerați că barierele în implementarea serviciului de vaccinare antigripală în farmacie dvs. sunt de natură: * Dropdown

Mark only one oval.

- ◯ Psihologică (personalul nu se simte confortabil, frică de răspundere, teama de reacțiile adverse post-vaccinare)
- ◯ Practică (lipsă spațiu, lipsă personal, cerere scăzută)
- ◯ Ambele

Skip to question 20

Factori economici

20. Considerați că serviciul de vaccinare ar fi profitabil pentru farmacia dvs.? * Dropdown

Mark only one oval.

- ◯ Foarte profitabil
- ◯ Moderat profitabil
- ◯ Puțin profitabil
- ◯ Deloc profitabil
- ◯ Nu pot estima

21. Care modalitate de plată pentru serviciul de vaccinare considerați că ar fi optimă? * Dropdown

Mark only one oval.

- ◯ Gratuită pentru pacient, decontată integral de CNAS/CJAS
- ◯ Parțial plătită de pacient, parțial decontată de CNAS/CJAS
- ◯ Integral plătită de pacient

22. Ce sumă (preț) credeți că ar **reflecta corect** valoarea acestui serviciu oferit de farmaciști? (costul vaccinului nu se include în această sumă) *

Mark only one oval.

- ◯ până în 20 lei/vaccinare
- ◯ 20-35 lei/vaccinare
- ◯ 35-50 lei/vaccinare
- ◯ Nu știu/Nu răspund
- ◯ Other: ______________________

23. În opinia dvs., cât de mare ar fi cererea pentru servicii de vaccinare în farmacia dvs. dacă ați oferi acest serviciu? *

Mark only one oval.

	1	2	3	4	5	
Foaı	◯	◯	◯	◯	◯	Foarte mare

24. Ați primit solicitări de la pacienți pentru servicii de vaccinare în farmacia dvs.? *

Mark only one oval.

- Da, frecvent
- Da, ocazional
- Foarte rar
- Deloc/Niciodată
- Nu știu/Nu răspund

25. În opinia dvs., unde preferă pacienții să se vaccineze *

Check all that apply.

- [ ] la medicul de familie
- [ ] la spital/clinică
- [ ] la farmacie
- [ ] nu știu/nu săspund
- [ ] Other: ____________________

Intenții viitoare

26. Intenționați să implementați serviciul de vaccinare antigripală în farmacia dvs. în viitor? * Dropdown

Mark only one oval.

- Da, în următoarele 6 luni
- Da, anul viitor
- Da, dar nu știu când (mai târziu)
- Nu
- Nu știu/Nu răspund

27. Ce v-ar determina să implementați serviciul de vaccinare în farmacia dvs.? (puteți selecta mai multe opțiuni) *

Check all that apply.

- [ ] Simplificarea procesului de autorizare
- [ ] Sprijin financiar pentru investiția inițială
- [ ] Cerere mai mare din partea pacienților
- [ ] Tarife (prețuri) mai mari pentru serviciul de vaccinare
- [ ] Compensarea serviciului de vaccinare de către CNAS
- [ ] Mai multe informații și instruire
- [ ] Other: ____________________

28. Ce măsuri ar putea lua autoritățile pentru a încuraja mai multe farmacii să ofere servicii de vaccinare?

29. Ce modificări legislative ar ușura procesul de vaccinare în farmacii?

30. Credeți că ar fi o lipsă a cererii serviciului de vaccinare antigripală de la pacienți în farmacia dvs? Dacă raspunsul este „DA”, care considerați că ar fi motivele?

31. Aveți alte comentarii sau sugestii referitoare la implementarea serviciilor de vaccinare în farmaciile comunitare?

Untitled Section

This content is neither created nor endorsed by Google.

Google Forms

Questionnaire on Barriers to the Implementation of Influenza Vaccination Services in Romanian Community Pharmacies

Research Purpose
This study aims to identify the barriers to implementing advanced pharmaceutical influenza vaccination services in pharmacies, with the goal of better understanding the challenges faced by professionals in the field.

Participation Criteria
This research is addressed exclusively to pharmacies that have NOT yet implemented influenza vaccination services.

Important: If you work in a pharmacy where influenza vaccination is already being performed, please do not complete this questionnaire.

General Instructions

- Please complete only one questionnaire per pharmacy.
- Estimated completion time: approximately 10–15 minutes.
- All responses are confidential and will be used exclusively for research purposes.

Ethical Considerations
This research has received approval from the Ethics Committee of the University of Medicine and Pharmacy (UMF).
Participation is voluntary; by completing this questionnaire, you express your consent to participate in the study.

Section 1: General Information

1. Type of Pharmacy

- Independent pharmacy
- Part of a national chain
- Part of a local/regional chain (operating in 2–3 counties)

2. Pharmacy Setting

- Urban
- Peri-urban (commune in a metropolitan area/suburb of a city)
- Traditional rural (commune, village)

3. Region in which the pharmacy operates *(Options: North-West, South-West Oltenia, South-Muntenia, West, South-East, North-East, Bucharest-Ilfov, Center)*

4. Approximate number of patients served daily (number of transactions/receipts)

- Under 100
- 100–300
- 300–500
- Over 500

5. Number of pharmacists working in the pharmacy (including part-time staff)

- 1
- 2–3
- 4–5
- More than 5

Section 2: Information on Influenza Vaccination Services in Pharmacies

6. Are you familiar with the legislation that allows pharmacies to provide influenza vaccination services?

- Yes, I am fully informed
- Yes, but I have limited information
- No, I am not aware of it

7. Have you been informed about the authorization process for providing vaccination services?

- Yes
- Yes, but I do not remember the details
- No

8. Have you considered implementing the influenza vaccination service in your pharmacy?

- Yes, I have analyzed this possibility
- Other pharmacies in our chain are already authorized to vaccinate
- Currently in the process of analysis
- No, I have not analyzed this possibility

Section 3: Resources and Infrastructure

9. Which of the following resources do you consider missing or insufficient in your pharmacy for the implementation of the vaccination service? (Select all that apply)

- Adequate physical space for a dedicated vaccination area
- Necessary equipment and materials (e.g., specialized refrigerator, protective gear)
- Sufficient qualified personnel for vaccine administration
- Financial resources for the initial investment
- Time to manage this additional service
- Other

10. Are there pharmacists in your team who have ALREADY COMPLETED the mandatory training course for vaccine administration?

- Yes/ No/ Don’t know

11. Are there pharmacists in your team who WOULD BE WILLING TO ATTEND the mandatory training course for vaccine administration?

- Yes/ No/ Don’t know

12. Estimate the total cost for setting up a vaccination space and acquiring the necessary equipment:

- Under 1,000 RON
- 1,000–3,000 RON
- 3,000–10,000 RON
- Over 10,000 RON
- Cannot estimate

Section 4: Regulations and Bureaucracy

13. How do you perceive the authorization process for vaccination services?

- Very complex and bureaucratic
- Moderately complex
- Relatively simple
- I am not familiar with the details of the authorization process

14. Which aspects of the authorization process do you consider significant barriers? (Select all that apply)

- Required documentation
- Space requirements

- Equipment requirements
- Time required to obtain the authorization
- Associated authorization costs

15. Are you concerned about the legal liability associated with vaccine administration (malpractice)?

- To a great extent
- To some extent
- To a small extent
- Not at all
- Don't know/Haven't thought about it

Section 5: Perceptions and Attitudes

16. To what extent do you believe that vaccine administration fits within the professional competencies of a pharmacist?

- Completely appropriate
- Partially appropriate
- Not appropriate for all pharmacists
- Not at all appropriate for pharmacists
- Don’t know/No response

17. What are the MAIN reasons why your pharmacy does not currently offer vaccination services? (Select up to 3 options)

- Lack of adequate physical space
- Lack of qualified personnel
- High implementation costs

- Complex authorization process
- Staff reluctance to administer vaccines
- Fear of legal liability
- Lack of reimbursement from the National Health Insurance House (CNAS)
- Lack of patient demand
- Competition from other vaccination providers
- Perceived lack of profitability

18. To what extent is there reluctance from pharmacy staff regarding vaccine administration? *(Scale from 1 to 5, where 1 is No reluctance and 5 is Very high reluctance)*

19. Do you consider the barriers to implementing influenza vaccination in your pharmacy to be primarily:

- Psychological (staff discomfort, fear of liability, fear of adverse reactions)
- Practical (lack of space, lack of personnel, low demand)
- Both

Section 6: Economic Factors

20. Do you believe that providing vaccination services would be profitable for your pharmacy?

- Very profitable/ Moderately profitable/ Slightly profitable/ Not at all profitable/ Cannot estimate

21. What do you consider to be the optimal payment method for the vaccination service?

- Free for the patient, fully reimbursed by CNAS/CJAS
- Partially paid by the patient, partially reimbursed by CNAS/CJAS
- Fully paid by the patient

22. What fee (price) do you believe would correctly reflect the value of this service provided by pharmacists (excluding the cost of the vaccine)?

- Up to 20 RON per vaccination
- 20–35 RON per vaccination
- 35–50 RON per vaccination
- Don’t know/No response

Section 7: Patient Demand and Future Intentions

23. In your opinion, how high would the demand for vaccination services be in your pharmacy if you offered this service? *(Scale from 1 to 5, where 1 is Very low and 5 is Very high)*

24. Have you received requests from patients for vaccination services in your pharmacy?

- Yes, frequently/ Yes, occasionally/ Very rarely/ Not at all

25. In your opinion, where do patients prefer to be vaccinated? (Select all that apply)

- At the family doctor
- At a hospital/clinic
- At a pharmacy

26. Do you intend to implement influenza vaccination services in your pharmacy in the future?

- Yes, within the next 6 months
- Yes, next year
- Yes, but I don’t know when (later)
- No
- Don’t know/No response

27. What would encourage you to implement the vaccination service in your pharmacy?

(Select all that apply)

- Simplification of the authorization process
- Financial support for the initial investment
- Higher patient demand
- Higher fees (prices) for the vaccination service
- Reimbursement of the service by CNAS
- More information and training

Section 8: Qualitative Feedback (Open-ended)

28. What measures could authorities take to encourage more pharmacies to offer vaccination services?
29. What legislative changes would facilitate the vaccination process in pharmacies?
30. Do you believe there would be a lack of demand for influenza vaccination from patients in your pharmacy? If "YES," what do you consider to be the reasons?
31. Do you have any other comments or suggestions regarding the implementation of vaccination services in community pharmacies?
